# Supplementary material for: Validation of reduced S-gene target performance and failure for rapid surveillance of SARS-CoV-2 variants
Source: PLoS One. 2022 Oct 3;17(10):e0275150. doi: 10.1371/journal.pone.0275150 (PMC9529109; doi:10.1371/journal.pone.0275150)
Supplement: S1 File — This Zip file contains the complete dataset used for all analyses, tables, and figures. Dates were adjusted by a random constant to assure deidentification. A data definition document is also included. (ZIP) [file pone.0275150.s001.zip › Covid Data Definition.pdf]

Validation of Reduced S-gene Target Performance and Failure for Rapid Surveillance of SARS-CoV-2 Variants

Data Definition

The file, Final COVID Dataset_Full.csv, contain data for every field for all samples included in the study (N: 374,469). All tables and figures in the manuscript are derived from these data.

1. Period – Days since first PCR sample was collected offset by a random constant number of days to assure deidentification
2. Ct_N – Ct value for N gene
3. Ct_S – Ct value for S gene
4. Ct_ORF1ab – Ct value for ORF1ab gene
5. Difference (N-ORF1ab) – Calculated difference between Ct values for N and ORF1ab
6. AVG of N and ORF – Calculated average Ct value for N and ORF1ab
7. S Gene Calculation – S gene Ct value – (Avg N and ORF1ab Ct values)
8. Amplification Category – Qualitative category for samples with normal S gene amplification, SGTF, and rSGTP
9. Category – Category for parent lineage
