# Supplementary material for: Validation of reduced S-gene target performance and failure for rapid surveillance of SARS-CoV-2 variants
Source: PLoS One. 2022 Oct 3;17(10):e0275150. doi: 10.1371/journal.pone.0275150 (PMC9529109; doi:10.1371/journal.pone.0275150)
Supplement: Attachment — Submitted filename: PLOS One Response.docx [file pone.0275150.s002.docx]

Response to Reviewers,

Thank you for the thorough review of our manuscript and the opportunity to submit a revised version. We have addressed each of the comments below and updated the manuscript where necessary.

- The general risk of this assay is in its inability to conclusively differentiate strains with the same deletion, as shown for Alpha and Omicron BA.1. however, it can differentiate variants with and without deletion mutants very effectively.
  - Response: This is a limitation of the approach that we have addressed in the manuscript discussion.
- It is still not very clear to me how the S-gene target performance reduction is measured. I understand you came up with a formula but what is the comparator to dictate the performance is reduced?
  - Response: The comparator to dictate the S-gene target performance is reduced is the performance of the N and ORF1ab gene targets within the same sample. The performance of all targets is measured by the amplification Ct value. There was a negligible difference between the Ct values of the N and ORF1ab-genes across all samples analyzed. The Mean and Median Ct Value difference between N and ORF1ab-genes for the lineages in this study has now been added to Table 2.
    - The Mean and Median Ct Value difference between N and ORF1ab-genes is less than 0.4 across all lineages. Once this was determined, we used the average Ct value of these two targets to compare against the S-gene Ct value.
    - The Mean and Median Ct Value difference between the S-gene and the average of N and ORF1ab-genes is less than 0.5 in Delta, Omicron BA.2, and Other lineages, indicating similar S-gene target amplification, or performance, in these lineages.
    - The Mean and Median Ct Value difference between the S-gene and the average of N and ORF1ab-genes is 5.08 and 5.31 for Alpha and 6.01 and 6.07 for Omicron B.1.1.529/BA.1/BA.1.1 lineages, respectively. The higher S-gene Ct Values in this sample set demonstrates weak amplification, or reduced performance, of the assay target in those samples where S-gene amplification occurred.
  - Response: Additionally, Table 1 and Figure 1 compares the frequency of rSGTP across all confirmed lineages in this study and further validates rSGTP in lineages with del69-70.
    - The proportion of samples with rSGTP was 0.07%. 0.05%, and 0.36% in Delta, Omicron BA.2, and Other lineages, respectively.
    - The proportion of samples with rSGTP was 17.07% and 7.47% in Alpha and Omicron B.1.1.529/BA.1/BA.1.1 lineages, respectively
- What if the primers/probe for S-gene are degraded or the sample has low viral load? How do you account for this?
  - Response: Degradation of the primers and probes would be observed in the positive controls. There are four positive controls on each RT-PCR run and all three genes, S, N, and ORF1ab, must amplify for the run and samples to pass. This has been added to the methods.
  - Response: To address samples with low viral loads, we specifically analyzed samples with an average N-gene and ORF1ab-gene Ct value < 30 to reduce the bias potentially introduced by these samples. Additionally, samples with lower viral load typically do not meet our quality criteria, or that which was set forth by the CDC, to make a definitive lineage call via our next-generation sequencing platform. These samples either result in incomplete sequencing, or result in a call for a less specific parent lineage. This is likely why the ”Other” category has an occurrence of SGTF and rSGTP that is higher than that found with Delta and BA.2. This is addressed in the methods and has been added as a limitation to the manuscript.
- The following statement in the discussion is very important in this study. I would recommend the authors to include this message concisely in the abstract as well "Our analyses also revealed a significant proportion of Alpha and Omicron B.1.1.529/BA.1/BA.1.1 samples with S-gene amplification (Table 1). For this reason, we believe that utilization of SGTF alone for early surveillance of variants with del69-70 would underestimate the actual changes in transmission of these variants".
  - Response: This has been concisely added to the abstract and manuscript.
- Please also highlight in the discussion, the limitation of the study and an explanation as in a scenario where any new emerging variants which does not carry the deletion at 69-70 codon. In addition, please comment on the samples which fail due to the low viral load, which might be below the cut off of the assay, but might work fine for N and ORF1ab.
  - Response: Statement of clarification and limitation added to the manuscript discussion.
- You have briefly indicated that this was done with a specific RT-PCR kit and in specific instrument. Do you anticipate change in results due to change of qPCR instruments?
  - Response: No meaningful changes would be expected due to a change of qPCR instrumentation. There could potentially be slight changes in the Ct values associated with each sample, but this would be minimally impactful and within the normal expected variance range of repeated analysis using this molecular technique. Additionally, other labs using the same reagent kit with different qPCR instruments have also observed SGTF and rSGTP in these lineages.
- Please also add a comment on the discrepant samples, that were missed by the assay.
  - Response: Table 1 indicates that our calculation for % of Samples with SGTF or rSGTP was unable to capture 100% of the confirmed Alpha (96.42%) or Omicron B.1.1.529/BA.1/BA.1.1 (99.65%) samples. The samples that were missed by our calculation were those where the Ct value of the S-gene was less than 4 cycles higher than the average of N and ORF1ab-gene targets. Figure 2 demonstrates the distribution of the differences between the S-gene Ct value and the average of N and ORF1ab-genes Ct value in Alpha and Omicron B.1.1.529/BA.1/BA.1.1 lineages. According to Poisson distribution, our calculation of rSGTP would capture ~90% or more of the Alpha and Omicron B.1.1.529/BA.1/BA.1.1 lineages with reduced S-gene target performance. This has been clarified in the results and discussion sections.
